# Supplementary material for: Content-rich biological network constructed by mining PubMed abstracts
Source: BMC Bioinformatics. 2004 Oct 8;5:147. doi: 10.1186/1471-2105-5-147 (PMC528731; doi:10.1186/1471-2105-5-147)
Supplement: Additional File 5 — The original Chilibot query results of the term "long-term potentiation (LTP)" and 22 other terms, limiting the latest references analyzed to the years 1990, 1995, 2000, and 2004. [file 1471-2105-5-147-S5.bz2 › chilibotAdditionalFile5/ltp2004/html/LTP_SYNAPTOPHYSIN.html]

 


 **LTP** and **SYNAPTOPHYSIN** 
  
Found 20 abstracts in PubMed,  **20 abstracts were retrieved and analyzed**.  


---

 Search Google  |
 PDF files only 
|  EDU domain only 

---

**Interactive relationship** (e.g. stimulation, inhibition, etc)

- The data suggest a role for  **synaptophysin**  in release and indicate that activation of tyrosine kinase and  **synaptophysin**  phosphorylation contribute to long term potentiation  [ **LTP** ]  perhaps by modulating glutamate release.  Ref: 9721920 Neuroreport, 1998
- Here, we show that late phase  **LTP**  L  **LTP**  is associated with a significant increase in numbers of synaptic puncta identified by  **synaptophysin**  and N cadherin, an adhesion protein involved in synapse formation during development.  Ref: 11086998 Neuron, 2000

- :-)

  **Parallel relationship** (e.g. studied together, co-existance, homology, etc.)

  - Both agents blocked induction of  **LTP**  and the increase in protein synthesis and  **synaptophysin**  synthesis which accompanied  **LTP** .  Ref: 9257941 Neuropharmacology, 1997
  - Here we investigate the role of  **synaptophysin**  in release and in expression of long term potentiation  [ **LTP** ]  in dentate gyrus.  Ref: 9721920 Neuroreport, 1998
  - Changes in protein synthesis and synthesis of the synaptic vesicle protein,  **synaptophysin** , in entorhinal cortex following induction of long term potentiation  [ **LTP** ]  in dentate gyrus an age related study in the rat.  Ref: 9257941 Neuropharmacology, 1997
  - We report that long term potentiation  [ **LTP** ]  was accompanied by increased endogenous glutamate release and increased tyrosine phosphorylation of  **synaptophysin** , but these changes were attenuated when long term potentiation  [ **LTP** ]  was inhibited by the tyrosine kinase inhibitor tyrphostin AG879 or by the NMDA antagonist D aminophosphonovalerate.  Ref: 9721920 Neuroreport, 1998
  - To explore further the role of the presynaptic terminal in long term potentiation  [ **LTP** ] , we have investigated changes in three synaptic vesicle proteins, synapsin, synaptotagmin and  **synaptophysin** , in control tissue and in tissue prepared from potentiated dentate gyrus 45 min and 3 h after induction of long term potentiation  [ **LTP** ] .  Ref: 7914357 Neuroscience, 1994
  - Evidence for a role for  **synaptophysin**  in expression of long term potentiation  [ **LTP** ]  in rat dentate gyrus.  Ref: 9721920 Neuroreport, 1998
  - The data presented here indicate that there was an increase in 35S methionine labelling of TCA precipitated proteins and 35S methionine labelling of  **synaptophysin**  in the ipsilateral entorhinal cortex 40 min after induction of  **LTP**  in dentate gyrus.  Ref: 9257941 Neuropharmacology, 1997
